# Lanoline

**Published:** 1887-07-30

**Authors:** 


### New Remedies.

### LANOLINE.

Whatever makes remedial agents more easy of application and more effective when applied is a distinct gain. Even grown-up people hate nasty physic, whilst children cannot and will not endure it. Everyone knows how difficult it is to prescribe for Dick, aged four, or his little sister, aged two-and-a-half. It often comes to this, that many drugs which are prescribed for adults without hesitation are practically unavailable for children, even when very much required, for the simple reason that the little rebels cannot be made to take them for love or money. Quinine is a case in point. It is often the very thing that children need above all others; as for example in certain fevers, or as a tonic, with iron, etc. But it is only here and there that you find a child who will endure what he considers the abominable bitterness of such physic. And we confess to a sneaking sympathy with the children. Quinine is nasty, and neither sherry, nor orange-wine, nor even Tinct. Ferri Perchlor. will make it nice.

Lanoline, the new base for ointment, which is prepared and sold by Messrs. Burroughs, Wellcome & Co., promises to be of signal service as a children's friend. It is both a doctor's and a patient's friend in many ways, being not only a curative agent in itself, but an admirable "base" for combination with all kinds of substances used in the formation of ointments and other outward appliances. Just now, however, we are anxious to emphasise it in the interest of the youngsters. With quinine, or iodide of potassium, for example, it forms an ointment as with other substances; but the great advantage of it is that it is so readily absorbed by the skin, that when the ointment is used the physiological action of the iodide or the quinine is obtained as decidedly as when either of the drugs is taken by the mouth, and that in a very short time. The advantages here are exceedingly obvious. Most medical men are by this time more or less familiar with the infinite variety of uses to which Lanoline may be put, and in which it will be really the best of all the similar substances which might be employed. It is, as everybody knows, a pure animal fat, and as its name implies, prepared from the sheep's wool. In the variety and extent of its application it probably surpasses any other substance which is known and used for like purposes.

